# A Walk in the Park: The Influence of Urban Parks and Community Violence on Physical Activity in Chelsea, MA

**DOI:** 10.3390/ijerph13010097

**Published:** 2016-01-04

**Authors:** Judy Y. Ou, Jonathan I. Levy, Junenette L. Peters, Roseann Bongiovanni, Jovanna Garcia-Soto, Rafael Medina, Madeleine K. Scammell

**Affiliations:** 1 Boston University School of Public Health, Boston University, Boston, MA 02118, USA; jonlevy@bu.edu (J.I.L.); petersj@bu.edu (J.L.P.); mls@bu.edu (M.K.S.); 2 Chelsea Collaborative, Chelsea, MA 02150, USA; Roseannb@chelseacollab.org (R.B.); Jgarciasoto@grassrootsonline.org (J.G.-S.); rafaelmariomedina@gmail.com (R.M.)

**Keywords:** built environment, physical activity, safety, parks, urban environment, Latino

## Abstract

Proximity to a park does not necessarily imply access or use, and the social environment may positively or negatively influence the positive intentions of the built environment. To investigate parks, park use and physical activity, and their associations with exposure to community violence, we interviewed residents (*n* = 354) of a densely populated urban community. Our findings indicate that proximity to any park is not associated with physical activity. However, proximity to the preferred park reported by residents to be conducive for physical activity (with walking paths, large fields, playgrounds for children and tennis courts) was associated with physical activity. Conversely, knowledge of sexual assault or rape in the neighborhood is inversely associated with every type of physical activity (park-based, outdoor, and indoor). Our findings suggest that improvements to the built environment (parks, green spaces) may be hindered by adverse social environments and both are necessary for consideration in the design of public health interventions.

## 1. Introduction

Physical activity protects against many health conditions, yet less than half of U.S. adults meet recommendations for physical activity [1]. Pedestrian walkways, parks, and green spaces in urban neighborhoods have been widely viewed as spaces that encourage physical activity, especially in low-income communities where residents often lack resources to use private exercise facilities [2]. However, proximity to parks by itself does not necessarily increase physical activity for residents of a given area [3]. Other social factors, such as crime rates, may result in residents feeling unsafe and unwilling to use local parks and walkways, even if such resources are available.

Previously reported findings on the association between parks and physical activity are mixed. One reason for this may be due to the variety of park types included in such studies [3]. Parks containing green spaces and trails for walking or running [4,5] or sporting facilities (e.g., tennis courts) would be suitable for adolescent or adult exercise, while parks consisting of small playgrounds designed for toddlers or children to use would not be conducive to adult exercise [6,7]. Many times, studies that rely on park counts do not take these differences into consideration. Weighting these different park types as equally useful to adults may obscure the relationship between park proximity and physical activity.

The relationship between community violence and physical activity is also unclear [8]. Community violence is a broad term that includes a wide range of events that could independently, or in combination, lead residents to feel unsafe [9]. Moreover, combining different types of neighborhood violence into a single violence measure may reduce the ability to discern the differential effects of individual types of violent events on physical activity [10]. The literature on identifying specific events that influence perceptions of safety or physical activity is sparse, although it includes a variety of measures such as perceptions of violence, perceptions of safety, and objectively reported rates of violent crime [8,9]. These different measures have different associations with physical activity, which appear to be driven more by perceptions of crime and safety than actual crime [11].

Improving physical activity is a public health priority for the City of Chelsea, MA. One survey of the city’s schools indicated more than half of all students were overweight or obese [12]. Chelsea’s age-adjusted mortality rate for coronary heart disease among adults, related to a lack of physical activity, is 70 percent higher than the state rate [13]. However, the 1.8 square-mile city contains over 20 parks [14], of varying sizes and exercise facilities, and Chelsea ranks among the best cities in Massachusetts for walkability [15]. Yet, this positive measure of the built environment may be offset by negative aspects of the social environment, with specific regard to crime. Chelsea’s reported violent crime rate is almost five times Massachusetts’ 2012 rate [16], and the presence of gangs is very strong in the public schools [17]. Identifying how residents use parks for physical activity, and whether community violence impedes physical activity in this community, could inform planning for this city and other urban populations.

This article explores the independent associations of physical activity with urban park access, as well as the association of knowledge of community violence and perceptions of personal safety with physical activity. We measure proximity to parks of various types, parks that appear suitable for adult physical activity, and parks residents report as usable.

## 2. Experimental Section

The Chelsea STAR (Science To Achieve Results) project, a community-university collaboration, is a cross-sectional study investigating residents’ health and environmental concerns [18]. Researchers and community members developed an interview guide including pre-validated and original questions that addressed local concerns.

Recruitment occurred between December 2011 and June 2013 via door knocking between 9 a.m. and 8 p.m. on weekdays and weekends. The study was publicized through cable television channels and flyers posted at community centers, clinics, homes, and local events. Eligibility criteria included: being 18 years of age or older, Chelsea residency for six months or more, English or Spanish language, and current residence in one of five census tracts near a designated port area. Interviews were conducted at participants’ homes, or in the community partner’s office in Chelsea. Geographic coordinates of participants’ homes were recorded. We obtained informed consent prior to each interview with approval from the Boston University Medical Campus Institutional Review Board.

We include questions from the interview guide that we used to measure park access and exposure to community violence in the Appendix.

### 2.1. Physical Activity

We asked participants if they engaged in any physical activity or exercise during the past month using a dichotomous response option (Yes/Any-PA, No/No-PA). If they indicated any type of physical activity, we asked open-ended questions about what participants do, and where they go for exercise.

Open ended responses were analyzed to identify the exact locations of physical activity, and assigned the categories of indoor physical activity (Indoor-PA), outdoor physical activity (Outdoor-PA), and park-based physical activity (Park-based PA). Indoor-PA identified participants that used indoor facilities (e.g., gyms). Outdoor-PA identified participants who reported outdoor activities (e.g., taking walks or running). Park-based PA (a sub-group of Outdoor-PA) identified participants who specifically stated they used parks for physical activity (e.g., playing tennis or running). No physical activity (No-PA) was the comparison group for all analyses.

### 2.2. Park Access

In addition to asking questions about physical activity, we asked a separate set of questions about parks and park use: the name of the park nearest their home, reasons why they use or do not use parks near their home, knowledge of other parks in the city, and places they recommend as good for taking a walk. Based on the response regarding the location of physical activity, we determined the park(s) used by each participant for physical activity. We also identified parks using the City of Chelsea’s park designations, excluding only one city-designated park which is a cemetery. The City classified each park as a Playground/Tot Lot, Sports, Walking, Sitting, or All facilities park, which indicated that the park contained all of the listed attributes.

We developed three proximity measures incorporating the park attributes and interview data. The first measured the distance between participants’ homes and the nearest park of any type (All parks). The second measured the distance to the nearest park with resources that adults would use (Parks with sports/walking facilities), and excluded parks classified only as playgrounds/tot lots or sitting areas. The third measured distance to the park that participants perceived as usable for physical activity (Resident-preferred park). Over 80 percent of participants that use parks for physical activity regarded this park as the most usable for physical activity, and a large number of participants also recommended this park as a good place to walk.

We divided distances from each of these park categories into quartiles, with the highest quartile (furthest distance) as the reference group.

### 2.3. Knowledge of Community Violence and Feeling Unsafe

We asked participants about their knowledge of specific types of violence in their communities using a modified version of the Exposure to Community Violence scale [19,20]. Participants reported whether they knew about one or more occurrences of a fight with a weapon, violent argument between neighbors, gang fight, sexual assault or rape, or robbery or mugging in their neighborhood in the last six months, and if they ever experienced violence against themselves or a member of their household while living in their neighborhood. We analyzed these events individually and with a total score, with one point per positive response and a maximum of six events [19,20]. Scores were divided into groups: no events (reference), one event, two events, or three or more events.

We also asked participants if they felt unsafe while walking alone during the day or at night. Participants responded using the options No problem/no opinion, Minor problem, or Serious problem [21]. We used No problem/no opinion as the reference group, with separate groups for the responses of Minor problem and Serious problem.

### 2.4. Individual Determinants

Participants reported their year of birth, sex, educational attainment, having children less than 18 years of age at home, race/ethnicity and occupation. If not working, we asked participants reasons why they currently were not working. We also asked participants who reported No-PA why they do not exercise. Participants who reported a temporary or permanent disability that prevented them from employment, and those who reported not exercising due to an injury, were counted as having an injury or impairment.

### 2.5. Statistical Analyses

Our objective was to determine which measures of park access and community violence were associated with physical activity, while controlling for key variables known to be individual determinants of behavior. Age, education, sex, ethnicity (Latino, not Latino), injury or impairment, having children younger than 18 years of age, work status (working, not working), and season were identified as variables that could be important in the models. Due to the high prevalence of our outcomes, we used robust log-linear regression models with a Poisson distribution to avoid biased effect estimates and confidence intervals, a method validated in previous cross-sectional analyses [22].

We used three different methods of variable selection to construct the most parsimonious final model. We examined associations between individual variables and outcomes for significance and ran stepwise selection (entry criteria = 0.10, stay criteria = 0.08) on a model including only significant variables. We then ran stepwise selection on a separate model that included all of the variables. We compared results from these two models to determine if variables recommended by the stepwise selection process were different from those that showed significant individual associations with the outcome.

We verified our model with LASSO (Least Absolute Shrinkage and Selection Operator), modified for use with dichotomous outcomes using a method described by Hastie *et al.* [23]. Our final model controlled for education, ethnicity, and injury or impairment. We highlight results that are significant (*p* < 0.05) and borderline significant (0.05 ≤ *p* < 0.1) while focusing on the magnitude of the odds ratios to provide insight about our findings [24]. 

## 3. Results and Discussion

### 3.1. Results

**Figure 1:**
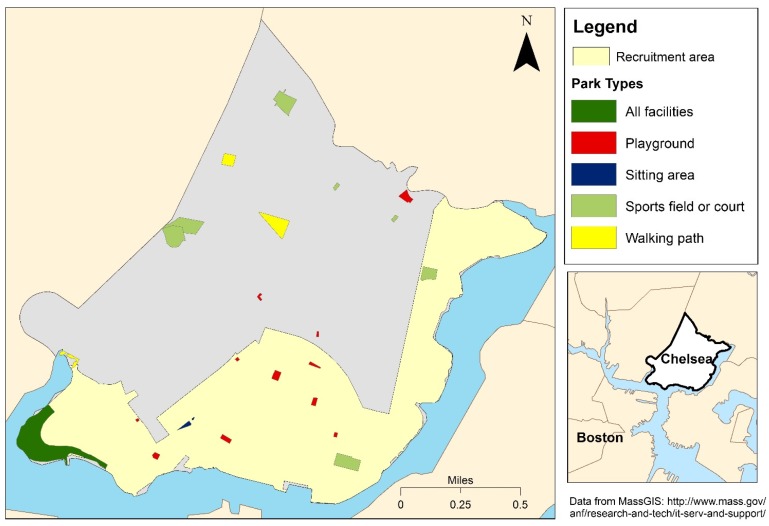
Locations and types of parks in Chelsea, MA.

We interviewed 354 residents, but excluded two from the final analysis due to missing data. The final study population includes 352 residents of Chelsea, MA. The majority of participants are middle-aged, female, Latino, and high school graduates (Table 1). Within our study population, less than 1% of participants are neither Latino nor non-Latino White, so we do not separately report race within our analyses. Sixty-four percent of our participants are not working, with 33 percent of the total participants reporting an injury or impairment that prevents employment or physical activity. Seventy-one percent report physical activity within the past month (Table 2), similar to results from the Massachusetts Behavioral Risk Factor Surveillance System [25]. The majority of participants who report physical activity in the past month report Outdoor-PA (39% of residents), meaning they use either parks, run or walk on sidewalks or trails, or other outdoor city resources for physical activity. Parks are very accessible in this environment, with participants living an average of 181 meters from a park of any type. Seventy three percent of participants live nearest to a playground/tot lot (Table 2). The locations and types of parks available in Chelsea, MA are shown in Figure 1.

**Table 1:** Study population characteristics.

Population Characteristics	Category	*n*	%
**Sex**	Female	239	68
	Male	113	32
**Ethnicity**	Not Latino	137	39
	Latino	215	61
**Education**	≥High school	231	66
	<High school	121	34
**Reported injury or impairment**	Yes	116	33
	No	236	67
**Children <18 years**	Yes	132	37
	No	220	63
**Age (years)**	18–44	152	43
	45–59	108	31
	60+	92	26

**Table 2:** Number and percent of reported physical activity (PA), local park types near home, and knowledge of community violence among the 352 study participants.

Physical Activity, Parks, and Community Violence	*n*	%
**Any-PA**	249	71
**Indoor-PA**	98	28
**Outdoor-PA**	139	39
**Park-based PA**	61	17
**Facilities in parks nearest home**		
Playground/Tot lot	256	73
Sitting area	49	14
Sports field/court or walking path	3	1
All facility types	44	13
**Knowledge of community violence**		
Gang fight	41	12
Fight with weapon	88	25
Robbery or mugging	93	26
Rape or sexual assault	20	6
Violent argument	116	33
**Personal experience with violence**	94	27
**≥1 Reported violent event (community or personal)**	215	61

Sixty-one percent of participants reported knowledge of, or experience with, one or more violent event occurring recently in their neighborhood (Table 2). Knowledge of a violent argument between neighbors was the most common type of event, followed by personal experience with any violent event. Participant knowledge of sexual assault or rape was the least common reported type of violent event in the community. Women may be more aware of sexual assault or rape, as women reported 16 of the 20 instances of a sexual assault or rape in the community.

Lower levels of physical activity are associated with participants who are Latino ethnicity, female sex, had less than a high school education, had children younger than 18 years, and who had an injury or impairment (Table 3). Latinos are significantly less likely to report any type of physical activity than non-Latinos. Participants that are 60 years of age or older are more likely to report Indoor-PA. There was no association between season and any physical activity measure, so season was not included in the final models.

**Table 3:** Individual odds ratios between demographic variables and physical activity (PA).

Demographics	Any-PA (*n* = 352)		Indoor-PA (*n* = 201)		Outdoor-PA (*n* = 242)		Park-Based PA (*n* = 164)	
	OR	95% CI	OR	95% CI	OR	95% CI	OR	95% CI
**Age**								
>60 years	1.20	0.89, 1.63	1.43 **	1.05, 1.95	1.18	0.89, 1.56	1.14	0.70, 1.87
45–59 years	1.11	0.82, 1.49	1.02	0.70, 1.48	1.19	0.93, 1.52	1.00	0.62, 1.61
18–44 years	1		1		1		1	
**Sex**								
Female	0.79 *	0.61, 1.03	0.68 **	0.52, 0.89	0.71 **	0.58, 0.87	0.49 **	0.34, 0.71
Male	1		1		1		1	
**Education**								
<High School	0.76 *	0.58, 1.00	0.46 **	0.31, 0.69	0.75 **	0.59, 0.96	0.37 **	0.21, 0.66
≥High School	1		1		1		1	
**Ethnicity**								
Latino	0.75 **	0.59, 0.97	0.60 **	0.46, 0.79	0.65 **	0.53, 0.80	0.55 **	0.38, 0.81
Not Latino	1		1		1		1	
**Children <18 years**								
Yes	0.83	0.64, 1.08	0.76 *	0.56, 1.03	0.79 *	0.62, 1.00	0.71	0.46, 1.08
No	1		1		1		1	
**Injury or impairment**								
Yes	0.76 *	0.57, 1.01	0.43 **	0.27, 0.69	0.76 **	0.59, 0.98	0.47 **	0.27, 0.81
No	1		1		1		1	

** Significant (*p* < 0.05); * Borderline significant (0.05 ≤ *p* < 0.1); **OR**: Odds Ratio; **CI**: Confidence Interval.

Associations between physical activity and park proximity differ based on the type of activity and type of park. Proximity to any type of park (All parks) is not associated with Any-PA, Indoor-PA, or Outdoor-PA (Table 4). Surprisingly, living near the Resident-preferred park is positively associated with Indoor-PA. We also find that the effect estimates for the association between the Resident-preferred park and Park-based PA do not differ greatly if participants live within a mile of the preferred park.

**Figure 2:**
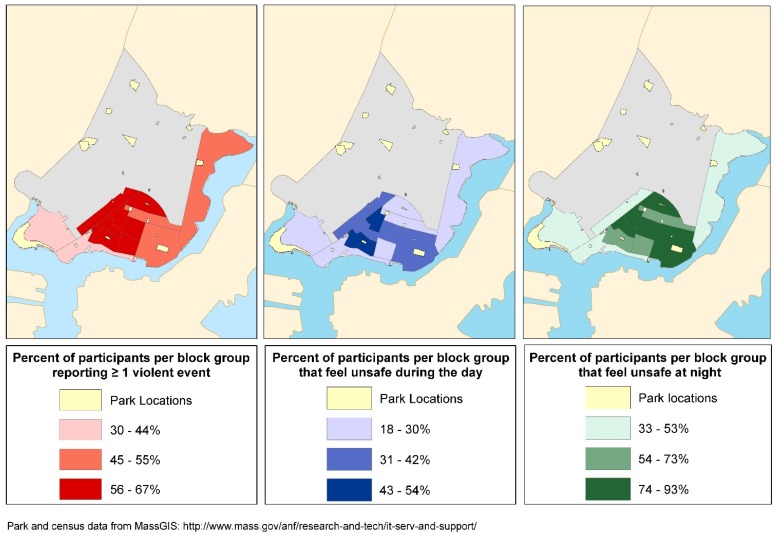
Park locations and perceptions of community violence and safety in Chelsea, MA.

Knowledge of individual violent events has a subtle association with physical activity (Table 5). Knowing about sexual assault or rape is the only type of community violence associated with physical activity, and is inversely associated with every physical activity outcome. Perceived safety does not show any association with physical activity (Table 6). Figure 2 shows the percent of participants within census block groups that are aware of one or more violent event, and report feeling unsafe during the day or night.

**Table 4:** Adjusted independent odds ratios between proximity to parks and physical activity (PA) **^a^**.

Park Categories	Any-PA (*n* = 352)		Indoor-PA (*n* = 201)		Outdoor-PA (*n* = 242)		Park-Based PA (*n* = 164)	
	OR	95% CI	OR	95% CI	OR	95% CI	OR	95% CI
**All parks**								
Quartile 1: 23–85 m	0.92	0.77, 1.11	0.94	0.66, 1.33	0.84	0.62, 1.15	0.88	0.47, 1.62
Quartile 2: 86–153 m	1.05	0.89, 1.24	1.19	0.85, 1.68	1.06	0.81, 1.38	1.58 *	0.99, 2.53
Quartile 3: 154–236 m	0.92	0.76, 1.12	0.93	0.63, 1.36	0.87	0.65, 1.18	0.87	0.47, 1.61
Quartile 4: >236 m	1		1		1		1	
**Parks with sports/walking facilities**								
Quartile 1: 75–461 m	0.99	0.82, 1.21	1.07	0.70, 1.65	0.96	0.71, 1.31	0.82	0.50, 1.34
Quartile 2: 463–638 m	1.03	0.84, 1.25	1.03	0.65, 1.63	1.06	0.80, 1.41	0.67	0.38, 1.19
Quartile 3: 640–835 m	0.95	0.77, 1.18	0.99	0.63, 1.57	0.97	0.70, 1.33	0.85	0.52, 1.38
Quartile 4: >835 m	1		1		1		1	
**Resident-preferred park**								
Quartile 1: 75–866 m	1.11 *	0.94, 1.32	1.45 **	1.02, 2.05	1.07	0.79, 1.44	2.42 **	1.12, 5.24
Quartile 2: 899–1269 m	1.00	0.80, 1.24	1.03	0.65, 1.63	1.00	0.72, 1.39	2.27 **	1.06, 4.87
Quartile 3: 1270–1606 m	1.10	0.90, 1.35	1.01	0.63, 1.61	1.24 *	0.93, 1.65	2.15 *	0.96, 4.81
Quartile 4: >1606 m	1		1		1		1	

**^a^** Adjusted for education, ethnicity, injury or impairment; ** Significant (*p* < 0.05); * Borderline significant (0.05 ≤ *p* < 0.1); **OR**: Odds Ratio; **CI**: Confidence Interval.

**Table 5:** Adjusted odds ratios between participant reports of community violence and physical activity (PA) **^a^**.

Community Violence	Response	Any-PA (*n* = 352)		Indoor-PA (*n* = 201)		Outdoor-PA (*n* = 242)		Park-Based PA (*n* = 164)	
		OR	95% CI	OR	95% CI	OR	95% CI	OR	95% CI
**Types of violent events **									
Gang fight	Yes	0.98	0.77, 1.24	0.95	0.61, 1.47	1.06	0.74, 1.52	1.34	0.84, 2.15
	No	1		1		1		1	
Fight using weapon	Yes	0.98	0.84, 1.16	0.92	0.67, 1.27	0.99	0.76, 1.29	0.89	0.57, 1.4
	No	1		1		1		1	
Violent argument	Yes	1.02	0.89, 1.17	0.99	0.75, 1.31	1.07	0.86, 1.34	1.33	0.92, 1.93
	No	1		1		1		1	
Sexual assault or rape	Yes	0.64 *	0.39, 1.04	0.52	0.23, 1.20	0.46 *	0.20, 1.05	0.35	0.10, 1.18
	No	1		1		1		1	
Robbery or mugging	Yes	0.99	0.86, 1.15	1.03	0.79, 1.35	0.99	0.76, 1.29	1.10	0.73, 1.64
	No	1		1		1		1	
**Personal experience with violence**	Yes	0.96	0.83, 1.13	0.96	0.69, 1.34	0.95	0.74, 1.21	1.17	0.80, 1.72
	No	1		1		1		1	
**Knowledge of multiple violent events**	1 event	1.04	0.9, 1.21	1.13	0.68, 1.86	1.11	0.87, 1.42	1.04	0.62, 1.76
	2 events	0.96	0.79, 1.16	1.04	0.60, 1.82	0.90	0.66, 1.23	1.20	0.74, 1.93
	≥3 events	0.99	0.80, 1.21	0.91	0.50, 1.67	1.04	0.74, 1.46	1.22	0.73, 2.06
	No events	1		1		1		1	

**^a^** Adjusted for education, ethnicity, injury or impairment; * Borderline significant (0.05 ≤ *p* < 0.1); **OR**: Odds Ratio; **CI**: Confidence Interval.

**Table 6:** Adjusted odds ratios between perceived safety and physical activity (PA) **^a^**.

Feeling Unsafe as a Problem	Any-PA (*n* = 352)		Indoor-PA (*n* = 201)		Outdoor-PA (*n* = 242)		Park-Based PA (*n* = 164)	
	OR	95% CI	OR	95% CI	OR	95% CI	OR	95% CI
**Feels unsafe during the day**								
Minor problem	0.97	0.82, 1.15	0.94	0.54, 1.63	0.97	0.74, 1.26	1.02	0.67, 1.53
Serious problem	1.10	0.88, 1.37	1.13	0.61, 2.08	1.14	0.79, 1.66	1.41	0.79, 2.53
No problem/No opinion	1		1		1		1	
**Feels unsafe at night**								
Minor problem	0.99	0.84, 1.16	0.99	0.59, 1.65	0.99	0.77, 1.29	0.82	0.51, 1.31
Serious problem	0.98	0.83, 1.16	0.96	0.58, 1.59	0.98	0.76, 1.28	0.93	0.57, 1.50
No problem/No opinion	1		1		1		1	

**^a^** Adjusted for education, ethnicity, injury or impairment; **OR**: Odds Ratio; **CI**: Confidence Interval.

### 3.2. Discussion

Our results support the idea that park access and reported knowledge of community violence influence physical activity but with some inconsistencies. Our analysis of qualitative data indicates that the majority of participants perceive one specific park as good for adult use, and most active participants use this Resident-preferred park. In Chelsea, this is the only park that contains large green spaces and walking paths, in addition to a soccer field, tennis courts, and playgrounds for children found in other parks. We were not able to assess if this preference was related to whether participants considered this park safe as we only asked participants about park safety in their own neighborhoods, although we note that the Resident-preferred park is in a low crime area of Chelsea (Figure 2). However, similar to previous findings, proximity to the nearest park of any type is not associated with physical activity [3]. Rather, proximity to the Resident-preferred park is positively associated with Any-PA, including either Park-based PA or Indoor-PA, or both. This finding supports previous literature reporting positive correlations between proximity to parks with green spaces and physical activity [26].

The effect estimates for the three quartiles measuring proximity to a Resident-preferred park and Park-based PA are very similar. The similarity suggests that adults in Chelsea will travel up to 1600 m from their residences to use parks perceived as usable. Since the average distance between a participants’ home and the nearest park of any type is under 200 m, this finding suggests that adults will bypass smaller parks and travel further to use the park with facilities better suited for their population and preferences. The widespread preference and awareness of the Resident-preferred park also support the idea that park accessibility is not defined solely by geographic proximity. Rather, perception of utility, awareness of parks, and geographic proximity all contribute to a nuanced definition of accessibility. This supports previous literature showing that use of neighborhood resources reflects resident perceptions of those resources [27].

We did not find a relationship between physical activity and perceived safety, or between physical activity and knowledge of multiple violent events, which are also reported as null in other studies [28]. These null associations support Foster *et al.*’s conclusion that aggregate measures of safety and violence mask true associations between specific types of violence and physical activity [9].

When we analyze reported knowledge of violent events individually, we identify an inverse association between knowledge about sexual assault/rape and any type of physical activity. The inverse association between knowing about sexual assault/rape and physical activity agrees with previous correlations between fear of, or experience with sexual assault and less physical activity [29,30]. Our findings also suggest that this is especially true for women [4]. Since women reported more sexual assault or rapes in their neighborhood than men, this specific type of community violence may impact physical activity among women more than men.

Beyond these findings, our study also identifies subpopulations at greatest risk for less physical activity. Women, participants with less than high school education, Latinos, having an injury or impairment, and having a child aged less than 18 years show consistent inverse associations with all measures of physical activity.

#### Strengths and Limitations

Our study’s strengths lie mainly in the use of qualitative data to inform creation of the park access and physical activity measures, along with detailed information regarding exposure to community violence. If we relied on conventional definitions of park access and physical activity used in previous studies [3], our analyses would show null results instead of the subtle relationships we identify between park types and the location of physical activity [31].

We are limited by the lack of information about the duration, frequency, and intensity of physical activity, which limits our ability to draw conclusions about the health implications of parks on changes in physical activity patterns. Our physical activity measures are also subject to potential recall bias.

Since this is a cross-sectional study, we are limited in our ability to explore causal mechanisms. We cannot determine whether the associations seen in our study are due to social causation (people exercise because they live closer to parks) or social selection (people who exercise chose to live closer to parks). We lack information on household income, and use education as a proxy measure. While this is common and typically captures socioeconomic status, it may not completely control for confounding given the influence of current income on ability to access physical activity resources.

In our study and others, larger parks and green spaces are located in higher economic status neighborhoods [32], whose residents may have more gym membership options. In our study, the highest percent of gym-users and those of higher economic status live near the preferred park, which may explain the positive association between Indoor-PA and proximity to the Resident-preferred park. This limitation further reinforces the complexities in drawing inferences from cross-sectional data.

Small numbers of participants who know about sexual assault or rape limit our ability to show statistically significant associations, although the trend in effect estimates and confidence intervals show consistent inverse correlations. Since our study population lives in one city, their park-use habits and preferences could differ from other populations. Chelsea is less than two square miles in size [33], which limits our ability to investigate how longer distances between residents’ homes and preferred parks might be associated with physical activity.

## 4. Conclusions

Urban parks can be valuable resources for physical activity, but parks should be built with the population’s needs in mind. The vast majority of participants prefer and use a park characterized by a wide variety of facilities and large green spaces with walking paths. We also found that knowledge of sexual assault or rape had a pronounced inverse association with any type of physical activity in this majority Latino population. In addition to shaping the built environment, safe social environments play a critical role in encouraging physical activity. Our study also reinforces the importance of qualitative data in environmental studies, which enables us to examine patterns in park preferences and how those preferences are associated with physical activity.

## Appendix

### A.1. Physical Activity

During the past month, other than your regular job, did you participate in any physical activities or exercises such as running, aerobics, dancing, or walking for exercise?

0. No 1. Yes

(If Yes) What do you do for exercise? (Note to interviewer: Get specifics: If running, where? If park, what park?) ______

(If Yes) Where do you go for exercise? ________

### A.2. Park Use

Do you know of any good places in Chelsea to take a walk?

0. No 1. Yes

(If Yes) Where? _______

Do you go to other parks in the city?

0. No 1. Yes

(If Yes) What parks? (1) ________ (2) ________ (3) ________

### A.3. Community Violence and Feeling Unsafe

#### A.3.1. Knowledge of Neighborhood Crime

To your knowledge, did any of the following occur in your neighborhood during the past six months:

A fight in which a weapon was used?     1. Yes 0. No

A violent argument between neighbors?   1. Yes 0. No

A gang fight?               1. Yes 0. No

A sexual assault/rape?           1. Yes 0. No

A robbery or mugging?           1. Yes 0. No

#### A.3.2. Personal Experience with Violence

While you have lived in this neighborhood, have you experienced violence, such as a mugging, physical fight, or sexual assault, against you or any member of your household anywhere in your neighborhood?

1. Yes 0. No

#### A.3.3. Feeling Unsafe [21]

The next set of questions is about the conditions of your neighborhood. I will ask you about something people may think is a problem in your neighborhood, and you respond with either (0) No opinion, (0) No problem, (1) Minor problem, (2) Serious problem:

Feeling unsafe in your home?

Feeling unsafe while out alone on the street during the day?

Feeling unsafe alone during the night?

Slow police response or police protection?
